# Structural Compensation and Participatory Approaches: A Way to Improve Access to Health Care for Undocumented Immigrants? A Scoping Review

**DOI:** 10.1007/s10903-025-01802-x

**Published:** 2025-11-18

**Authors:** Camille Guiheneuf, Marie Dauvrin

**Affiliations:** 1 UCLouvain-Institute of Health and Society (IRSS), Brussels, Belgium; 2 Belgian Health Care Knowledge Centre (KCE), Brussels, Belgium

**Keywords:** Undocumented immigrants, Health services accessibility, Community-based participatory research, Participatory research, Non-governmental organizations, Structural compensation

## Abstract

In Europe, non-profit organisations provide health care to undocumented immigrants (UDM), filling the gaps left by national healthcare systems: this approach is referred as “structural compensation”. Integrating beneficiaries in developing interventions can be a source of effectiveness, but evidence shows that UDM involvement is difficult. This scoping review aimed to identify best practices related to participative approaches in structural compensation interventions targeting UDM. Four electronic databases were searched. Nine studies, which met the final inclusion criteria, reported a variety of health interventions. Findings showed a limited but diverse use of participatory approaches. Furthermore, the structural compensation organisations could be facilitators to support the participation of UDM. Further research is needed to better understand structural compensation and how it is used in practice. This will help develop an appropriate response to participative approaches with UDM.

**Supplementary Information:**

The online version contains supplementary material available at 10.1007/s10903-025-01802-x.

## Background

Over the last decades, a growing body of literature stressed the need for more inclusive and responsive healthcare systems for migrants, including undocumented immigrants (UDM) [1]. Migration has been acknowledged as a social determinant of health [2], likely to negatively affect physical and mental health. Moreover, UDM are particularly at risk of being exposed to hazardous and harmful circumstances (e.g. unstable housing, food insecurity, violence, lack of medical insurance), jeopardising their health and well-being [3]. However, despite international agreements guaranteeing access to health care as a universal right for all, regardless of legal status, UDM still face numerous barriers [4–8]. At policy level, across Europe, access through national healthcare systems highly varies from health care limited to vital emergencies to a health care coverage similar to that of nationals [9]. However, even in countries where access is granted, UDM still face access problems because of institutional and individual barriers [8].

Consequently, to counterbalance these barriers, non-profit organisations have taken initiatives to improve health access for UDM. This approach has been described as “structural compensation” as it aims to fill the gaps in the healthcare systems by compensating for limited access to health care for vulnerable populations [9–12]. This approach was developed by Non-Governmental Organisations (NGO) to support groups in vulnerable situations, and has been used to address ethical dilemmas relating to health [9, 10]. Currently, these interventions often arise from citizens and/or health care professionals’ observations of the unmet needs of UDM, particularly in crisis contexts [13]. Structural compensation interventions can take different shapes at the infrastructure or the workforce level (e.g. provision of health care or health information, navigation through the healthcare system) [14–16] and can be implemented by different actors (NGO, citizens’ organisations, etc.).

In this context, including a participatory approach to the interventions can enhance their overall effectiveness. Participatory approaches aim to enhance access to health care, quality of care, healthcare utilisation, and health outcomes by involving participants at various stages of the intervention process [17–19]. Applied to health research, these approaches contribute to the establishment of research priorities by acknowledging the norms, cultures and expectations of participants [20]. Thus, the research outcomes will be perceived as more useful and rooted in needs of public and professionals [21, 22]. It has a positive impact on treatment adherence, patients’ and health care professionals’ satisfaction [18, 21, 22], health care improvement and healthcare system organisation (e.g. a better inclusion of marginalised people in the healthcare system) [17, 23]. Getting people involved contributes to their health empowerment [21, 24–26]. In addition, the interventions are more concrete, effective, relevant and more likely to be implemented in the long term [27–29].

However, the majority of participants in participative interventions do not experience socio-economic difficulties [26, 30, 31], and are able to commit time [32]. This combination of circumstances rarely occurs in vulnerable populations, and they are often excluded from participatory approaches [26, 33]. Additionally, UDM are often marginalised [27, 28, 34] and, therefore, more difficult to include in participatory approaches [26].

However, not including UDM in participatory approaches can negatively impact the effectiveness of these interventions. Indeed, involving beneficiaries contributes to a better understanding of their expectations or needs according to their personal circumstances. This is crucial to obtain appropriate, relevant and acceptable interventions. Not including UDM as actors of the development of the intervention comes with a risk of wasting resources because of inappropriate interventions that could lead to poor health outcomes [35, 36]. As NGO often depend on public subventions and charity money, they also have to be accountable for the impact of their actions, hence the need for interventions matching the needs of the public they serve.

To the best of our knowledge, no previous study has investigated how structural compensation interventions include participatory approaches to improve access to health care for UDM. This scoping review aimed to analyse the existing literature on structural compensation for UDM, identifying participative approaches and the factors supporting or impeding their implementation. This scoping review was also an opportunity to critically reflect on missed opportunities within structural compensation interventions to incorporate participation. It may be of interest to those already involved in structural compensation interventions and to those who wish to increase the involvement of UDM in the design of such interventions.

## Methods

The Arksey and O’Malley methodology was employed, comprising five steps [37]. The scoping review was conducted following the PRISMA Extension for Scoping Reviews (PRISMA-ScR) guidelines [38].

### Identifying the Research Question

The research question is defined as follows: How are participatory approaches included in structural compensation interventions aiming to improve access to health care for undocumented immigrants in Europe?

For this scoping review, an “intervention” is defined as any action, project, or program aiming to improve healthcare access.

### Search Strategy

Eligible studies were identified across four databases: Scopus, PubMed, CINAHL and Embase. An initial limited search of PubMed was undertaken to identify keywords. To capture all eligible studies, we followed a search strategy that allows using both MeSH terms and free text. Only PubMed database used MeSH terms. The search strategy was adapted for each database included. An information specialist helped the authors to build the search strategy and adapt it to each database. Table 1 provides an example of the search strategy used for the PubMed database (Full search details are available in Supplementary File 1).

**Table 1:** Search strategy for pubmed database

Search strategy	Date of search	Results
(“Undocumented Immigrants“[mh] OR “undocumented migrant*“[tiab] OR “undocumented immigrant*“[tiab] OR “undocumented foreign-born“[tiab] OR “undocumented patient*“[tiab] OR “undocumented worker*“[tiab] OR “undocumented migration*“[tiab] OR “undocumented immigration*“[tiab] OR “unauthorized migrant*“[tiab] OR “unauthorized immigrant*“[tiab] OR “unauthorized foreign-born“[tiab] OR “unauthorized patient*“[tiab] OR “unauthorized worker*“[tiab] OR “unauthorized migration*“[tiab] OR “unauthorized immigration*“[tiab] OR “irregular migrant*“[tiab] OR “irregular immigrant*“[tiab] OR “irregular patient*“[tiab] OR “irregular worker*“[tiab] OR “irregular migration*“[tiab] OR “irregular immigration*“[tiab] OR “irregular labor“[tiab] OR “unregistered migrant*“[tiab] OR “unregistered patient*“[tiab] OR “unregistered worker*“[tiab] OR “illegal migrant*“[tiab] OR “illegal immigrant*“[tiab] OR “illegal worker*“[tiab] OR “illegal migration*“[tiab] OR “illegal immigration*“[tiab] OR “illegal labor“[tiab] OR “illegal labour“[tiab] OR “Undocumented Alien*“[tiab] OR “Undocumented Latino Immigrant*“[tiab] OR “Undocumented Latinx Immigrant*“[tiab] OR “Undocumented African Immigrant*“[tiab] OR “Undocumented Adult*“[tiab] OR “illegal alien*“[tiab] OR “illegal migrant worker*“[tiab] OR “undocumented migrant worker*“[tiab] OR “Undocumented Immigrant“[tiab:~2] OR “Undocumented Immigrants“[tiab:~2]) AND (“Health Services Accessibility“[mh] OR “Health Services Research“[mh] OR “Insurance Coverage“[mh] OR “Health Services Accessibility“[tiab] OR “Access to Health Services“[tiab] OR “Access to Health Care“[tiab] OR “Accessibility of Health Services“[tiab] OR “Availability of Health Services“[tiab] OR “Health Services Availability“[tiab] OR “Access to Care“[tiab] OR “Access to Cares“[tiab] OR “Program Accessibility“[tiab] OR “Access to Therapy“[tiab] OR “Access to Therapies“[tiab] OR “Access to Treatment“[tiab] OR “Access to Treatments“[tiab] OR “intervention accessibility“[tiab] OR “health access“[tiab] OR “health accessibility“[tiab] OR “healthcare access“[tiab] OR “healthcare accessibility“[tiab] OR “health care access“[tiab] OR “health care accessibility“[tiab] OR “health service access“[tiab] OR “health services access“[tiab] OR “health service access“[tiab] OR “health service accessibility“[tiab] OR “access to health care“[tiab] OR “Access to Health Service“[tiab] OR “access to healthcare“[tiab] OR “access to health“[tiab] OR “care access“[tiab] OR “care accessibility“[tiab] OR “Health Services Research“[tiab] OR “Health Care Research“[tiab] OR “Healthcare Research“[tiab] OR “Action Research“[tiab] OR “Health care coverage “[tiab] OR “Health Care Delivery“[tiab] OR “access to health insurance“[tiab] OR “Universal Health Insurance“[tiab] OR “Universal Coverage“[tiab] OR “Health insurance“[tiab] OR “Insurance Coverage“[tiab] OR “Insurance Status“[tiab] OR “accessible health care“[tiab] OR “accessible health services“[tiab] OR “accessible healthcare“[tiab] OR “health service delivery“[tiab] OR “accessing healthcare“[tiab] OR “continuum of care“[tiab] OR “health coverage“[tiab] OR “healthcare provision“[tiab] OR “health care provision“[tiab] OR “health service organization“[tiab] OR “health services organization“[tiab] OR “health service organisation“[tiab] OR “health services organisation“[tiab] OR “Program Access“[tiab] OR “Intervention Access“[tiab] OR “Therapy access“[tiab] OR “Treatment access“[tiab])	16/06/2024	807

### Selection Process

**Fig. 1:**
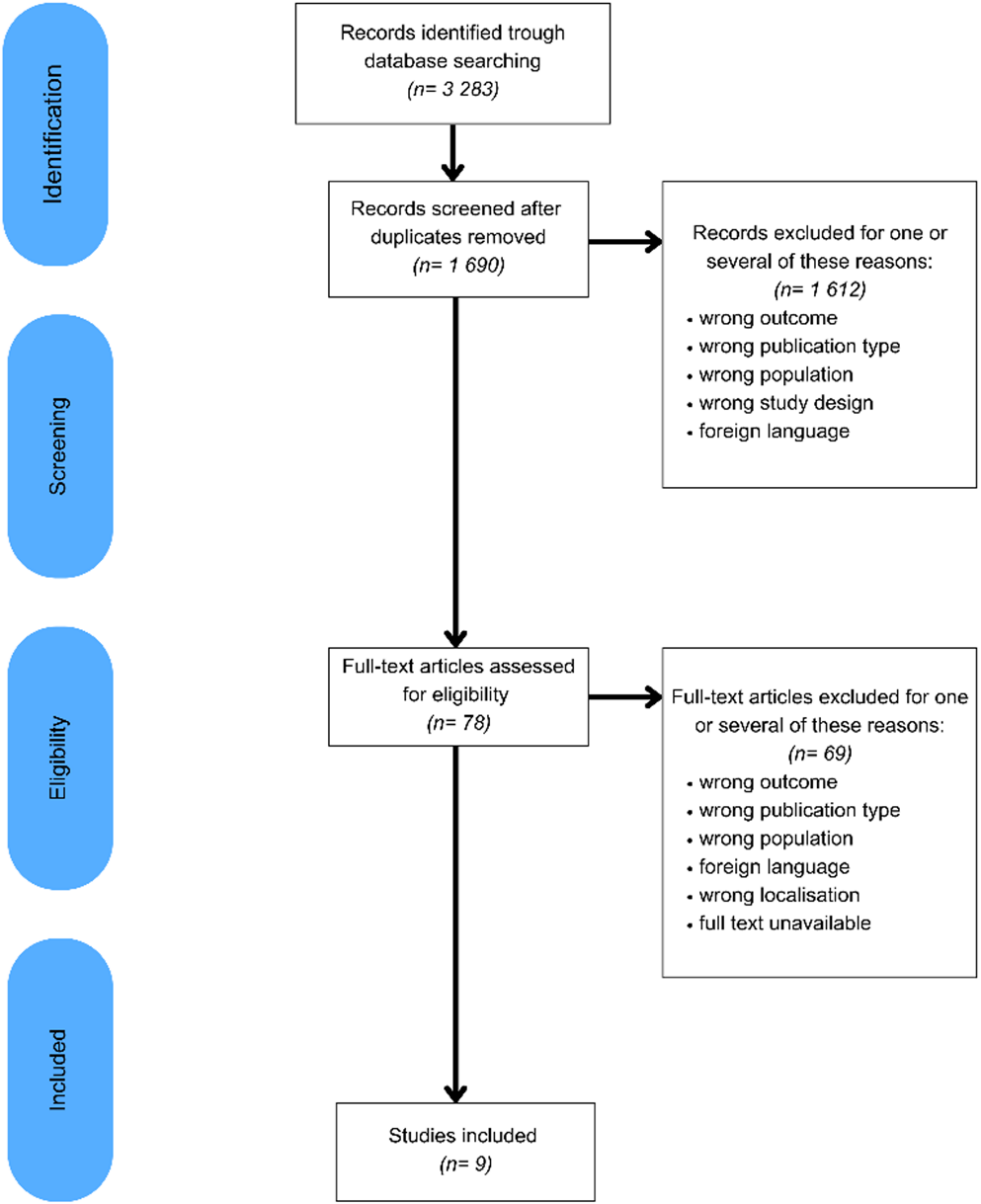
Flow diagram of the study selection process

Figure 1 presents the flow chart of the selection process. All identified citations in each database were gathered into Endnote™21 before being screened via the web-based application Rayyan to remove duplicates. The two authors independently reviewed titles and abstracts based on the inclusion and exclusion criteria (Table 2).

**Table 2:** Inclusion and exclusion criteria applied in the review

Inclusion criteria	Exclusion criteria
Citations reporting health interventions aimed at improving healthcare access for UDM	Commentary; Study protocol; Conference proceedings
No time restriction	No abstract available
Limited to European countries	
French, English	

Due to the invisibility and marginalisation of undocumented migrants, which pose challenges to data collection and research publication, no date restrictions were applied in order to capture the broadest possible range of relevant studies.

Then, the two authors independently reviewed the full text of selected citations. At this stage of the reviewing process, disagreements between the reviewers during the selection process were resolved through discussion.

### Data Extraction

Extracted data were included in an Excel file: the studies’ characteristics, the interventions’ characteristics and the participatory approaches’ characteristics. The two authors independently extracted data from three studies and completed the data-charting form to ensure the validity and comprehensiveness of each category (see Supplementary File 2 for the list of each topic investigated). The file was adapted to be used as a framework for the data analysis. Data extraction and categorisation were regularly discussed to support the consistency of the interpretation of the data. Participation was defined as the application of democratic values and social justice in health interventions [39] by providing people with the possibility of having control over their health [40]. Consequently, in this review, participation was operationalised by identifying elements of the intervention showcasing a process of distributing power through direct and concrete meaningful involvement in decision-making, problem definition and action [41, 42].

### Summarizing and Reporting the Results

The narrative synthesis process is intended to provide a comprehensive summary of the results, extending beyond a simple description. It is “a textual approach to the process of synthesis to ‘tell the story’ of the findings from the included studies.” [43], p. 5.

First, the results were organised into groups (type of intervention, context, target population, outcomes, etc.) using textual descriptions from the included studies, in order to highlight important aspects. As the grouping process progressed, common themes emerged, providing a structured framework for reporting the results.

## Results

### Study Selection

From the 78 articles screened for full text, nine met the inclusion criteria. Six quantitative studies [44–48] and three qualitative studies [50–52] were included. Seven studies included UDM as the unique recipients of the intervention. Two studies did not exclusively include UDM, but based on the assessment of both authors, UDM constituted a sufficient majority of the sample for the findings to be considered reflective of UDM-specific characteristics [45, 49] (Fig. 1).

**Table 3:** Descriptive characteristics of interventions studied included in the scoping review (*n* = 9)

First Author	Country	Health issue	Aim of the intervention	Target group	Setting
Eick et al. (2022)	Norway	Sexual and reproductive health	To provide maternal and perinatal health care	Undocumented immigrant women	NGO clinics
Bousmah et al. (2023)	France	Sexual and reproductive health	To provide a health-related empowerment intervention on access to health coverage	Undocumented adult and elderly immigrants from sub-Saharan Africa	Place of residence of UDM (outreach)
Knudtzen et al. (2022)	Denmark	No specific health problem	To provide free health care, including an outreach programme	Undocumented immigrant women (90.7% of the participants) – mainly sex workers	Free healthcare clinic and place of residence of UDM (outreach)
Lambert (2018)	Belgium	Oral dental health	To increase awareness and attendance of UDM in dental care treatments by involving community health workers	Undocumented immigrants of all ages	Dental practices in the mainstream healthcare system
Castaner et al. (2022)	Denmark	Sexual and reproductive health	To provide sexual and reproductive health care	Undocumented immigrant women	NGO healthcare centres One healthcare centre embedded in a public hospital
Mason (2016)	Spain	No specific health problem	To use a novel strategy using linking social capital to provide healthcare access	Undocumented adult and child immigrants	Church
Morisod et al. (2023)	Switzerland	Vaccination (COVID-19)	To implement a COVID-19 vaccination program, including a communication campaign	Undocumented adult immigrants	Regional academic centre of general medicine and public health
Rosa-Hezode et al. (2019)	France	Screening	To implement a hepatitis C virus systematic screening programme including screening, management, follow-up and treatment	Undocumented adult immigrants and asylum seekers	Primary care centres
Van Midde et al. (2020)	Netherlands	Oral dental health	To provide dental treatments by a voluntary dental network	Undocumented adult immigrants	Dental clinics in the mainstream healthcare system

### Characteristics of Interventions

Table 3 presents the health issues addressed by the interventions, their aims, the target groups and the settings where the interventions took place.

Interventions consisted in provision of preventive care and/or health promotion in eight studies [45–52], medical care in five studies [44, 46, 47, 50, 52] and other types of service such as translating services [44, 47, 48, 50], administrative support [49, 52] or emotional support [50].

Seven interventions included a formal referral to the national healthcare system [44–47, 50–52], reflecting an attempt to remedy the insufficient coverage by or use of existing services. In three interventions, the referral was supported by an assessment of needs and orientation to the healthcare system [47, 51, 52]. Two interventions had no referral procedure, but it was not relevant in the design of the intervention [48, 49].

Information about health outcomes has been retrieved during the data extraction and analysis. However, these findings were not mentioned in this paper because they go beyond the research objective. In addition, a different type of methodology would be required to assess the interventions’ effects, particularly to appraise the complexity of the interventions and the diversity of socio-political contexts of the different settings.

### Operationalisation of the Participatory Approaches in Structural Compensation Interventions

This scoping review aims to analyse the literature on structural compensation for UDM health access, focusing on participatory approaches and their characteristics throughout the intervention process. These approaches are designed to improve access to health care (e.g. quality, utilisation). An overview of stakeholders’ participation according to the stages of the interventions is provided in Table 4. The following subsections present findings relating to the stages of the intervention process, including initiation, design, implementation, data collection, analysis, and adaptation.

**Table 4:** Participation of stakeholders regarding the stages of the interventions

Authors	Stakeholders	Intervention stages
Eick et al. (2022)	UDM	Design; analysis and adaptation
	Health care professionals (NGO)	Design; implementation; analysis and adaptation
Bousmah et al. (2023)	UDM	Design
	Health care professionals	Implementation
	Research team	Initiation; Design; implementation
	Community-based organisations	Design; implementation
Knudtzen et al. (2022)	UDM representatives	Design
	NGO	Implementation
Lambert (2018)	Community-based organisations	Initiation; design; implementation
	Health authorities	Design
	Health care professionals (NGO)	Design; data collection
Castaner et al. (2022)	UDM representatives	Design
	Health care professionals (NGO)	Implementation; analysis and adaptation
Mason (2016)	Community-based organisation	Implementation
	UDM	Analysis and adaptation
Morisod et al. (2023)	Community-based organisation	Implementation
	Health authorities	Implementation
	Health care professionals	Implementation
Rosa-Hezode et al. (2019)	UDM representatives	Design
Van Midde et al. (2020)	UDM	Initiation; data collection; analysis and adaptation
	Health care professionals	Analysis and adaptation

### Initiation Stage

The initiation stage refers to the source of the idea for the intervention. Overall, the projects did not originate with the UDM themselves, except for the intervention described by Van Midde et al. [52]. In this study, a religious community, which included UDM as active members, identified the health issues and initiated the intervention [52]. In Bousmah et al., the interventions were initiated by a research team [45], and in Lambert by community-based organisations [47]. The initiation stage was informed by scientific/grey literature [44, 45, 48], public health data [51] or health care professionals’ knowledge [50].

### Design Stage

The design stage refers to the development and adaptation of the intervention to the specific needs of the UDM. UDM contributed to the design of two interventions [44, 45]. In Bousmah et al., the design arose from a participatory process which included a research team, two community-based organisations and UDM [45]. In Eick et al., NGO health care professionals and a group of UDM were invited to share their understanding of the vulnerabilities they faced [44]. These two studies provided no further information on how participation was facilitated.

In three interventions, the following actors were included as proxies to adapt the design to UDM: the local voluntary-based organisation [47], health authorities [48], or NGO [52]. Three studies did not specify this information, but actors included in the design stage probably tended to defend the health interests of UDM [40, 46, 49]. Despite not involving UDM, some interventions were adapted to facilitate their access to health care: tailored therapeutic patient or health education [49, 51], outreach activities in UDM living spaces [45, 46], translated information and extended opening hours [48]. However, some interventions faced constraints preventing UDM from accessing the services, such as restricted opening hours due to the limited availability of resources [44, 46, 51], and restrictive legal frameworks (e.g. healthcare practitioners have to mobilise an informal network to provide sexual and reproductive healthcare) [50].

### Implementation Stage

The implementation stage refers to leadership, coordination and execution of the intervention in the field. UDM did not contribute to the implementation in any of the studies. This was done by NGO [44, 46, 50, 52], community organisations [47, 48, 51], researchers [45], healthcare centres and health authorities [48]. Those delivering the intervention were mostly health care professionals (e.g. GPs, nurses, midwives). Implementation was facilitated by the cultural competencies of the staff [45, 50],, their experience with people in vulnerable situations [48] or a shared background with the patients they served [45, 50]. For example, in the study of Castaner et al., one of the professionals was a former sex worker, allowing her to create trust, facilitate communication and break cultural barriers when discussing sex and contraception with UDM [50].

The inclusion of community-based organisations [45, 47, 48, 51] was identified as an implementation facilitator. In Lambert’s study, implementation was facilitated by community health workers spending time in the community and by social workers who received a community health training conducted by two academics (the community-based organisation was not involved in the training) [47]. Community-based organisations, social workers or NGO could act as UDM representatives or establish connections between them and the various of stakeholders involved in interventions. The potential role of these organisations as facilitators was not addressed in the analysed studies. However, across the studies [45, 47, 48], establishing collaboration with community-based organisations appeared as a key facilitator to support the implementation of structural compensation interventions. As community-based organisations are identified as reliable partners by UDM, they are a relevant starting point for initiating and implementing participatory interventions with UDM.

### Data Collection

The data collection stage refers to the need to obtain feedback to adapt the interventions. UDM did not collect such data, and only one study directly questioned the UDM about their perspectives and expectations relating to the intervention [52]. These data were triangulated with those of the professionals involved in the intervention, but no further details were provided about how this feedback was included in the intervention. Castaner et al. also used interviews to collect data, but only with health professionals, to explore tactics employed to meet sexual and reproductive health care needs [47]. Three studies relied on administrative data to assess and adapt the intervention: number of recipients [44, 48, 51], number of contacts with the service (frequency) and length of the care pathway [45, 46], type of consultation [46] or number of missed appointments [47].

### Analysis and Adaptation

The analysis and adaptation stage refers to the interpretation of the data and the adaptation of the intervention to the specific needs of the UDM. Three interventions included UDM and health professionals at this stage [44, 51, 52]. In the intervention of Eick et al., preliminary results were discussed with NGO staff and UDM, but no further information was reported about the impact of these discussions on the intervention [44]. Van Midde et al. triangulated the experiences of health professionals and UDM regarding dental care: this helped to identify the need for guidelines for professionals, as well as the need to integrate patient experiences into the evaluation of the intervention [52]. In the third intervention, UDM leaders were involved in discussions about their perception of the needs of the UDM, the adequacy of the services provided and the barriers when accessing these services. Regular meetings with local NGOs and humanitarian organisations (as representatives of UDM) to assess the utilisation of resources and the adequacy of the strategies aiming to reduce the barriers to access previously identified [51].

## Discussion

This scoping review examined the use of participatory approaches in structural compensation interventions for UDM healthcare access.

Nine studies about structural compensation interventions in Europe were identified. Only one study explicitly mentioned the use of a participatory approach but, from our analysis, it emerged that four of the studies included a participatory component with UDM in at least one stage of the interventions. This leads us to believe that interventions can be participatory without being recognised as such by their authors. This finding somehow nuances the apparent poor participation of UDM in the development of the interventions aiming to improve their access to health care. However, some studies may describe a participatory process involving no real participation or only token participation of UDM, meaning that the UDM are invited to participate by the developers but are not listened to or are not supported in their capacity to influence the development of the interventions [53]. Overall, migrants are rarely involved in health research and current practices tend to exclude them and use tokenistic practices [54]. The systematic review of Rustage et al. illustrates the feasibility of including UDM’s participation in health interventions. This study has reported 30 instances of proxies involvement, 46 instances of active participation and 24 instances of indirect participation of migrants. Active, indirect participation or proxies involvement would help interventions about UDM health access to support, as much as possible, this population’s health needs. However, failing to establish equitable partnerships may limit the reliability of the findings [53].

UDM were more involved in the design and analysis/adaptation stages and absent from the implementation stage, which is consistent with the limitations to participation faced by UDM. It might also reflect the relative ease or difficulty of participation in a given stage, as design and adaptation concerns tangible and visible activities that can be addressed during a one-shot consultation, while initiation, for instance, involves more activities that may require several meetings to develop a well-defined project. Involving UDM in the implementation also raises legal and ethical concerns: employing an UDM can be against the law. Furthermore, requiring volunteer work of persons in vulnerable situations does not promote social justice, which is at the core of the participative approaches. To remain meaningful, participative approaches need to respect ethical and deontological guidelines, with a view of preserving the integrity and the well-being of UDM. As mentioned by Lander et al., uncertainty persists about how to engage non-academic stakeholders [55]. There can be no “best practice” for participation, as this is always context-dependent and rooted in specific sociocultural contexts [27]. These findings prompt the further question of whether it is advisable to include UDM at all stages of structural compensation interventions.

NGO, community-based organisations or research teams were involved as participants at several stages of the interventions. Their involvement could be identified as a proxy (or a representative), and for setting up interventions on behalf UDM (e.g. adaptation of intervention, voicing collective concerns). Involving such proxies is a valuable option when a participatory intervention raises ethical issues [27]. For instance, they are likely to have specific abilities such as displaying arguments or negotiating and establishing strategic alliances [56–58]. The representation of UDM could guarantee the intervention’s efficiency [59]. However, involving a representative has its limits. Patients’ perspectives may be misguided, and the distribution of resources based on the representatives’ perspectives may deviate from UDM’ actual needs [60]. These limits are not UDM-specific, but could have a stronger impact on their health. UDM are marginalised and, thus, have fewer opportunities to influence the decisions that are taken on their behalf. This raises the question of how to empower representatives to be able to speak on behalf of others [61], and of their relationship with those who they represent [59]. Ultimately, the question that is raised by being a representative is “*what should be done to ensure their [migrants] views are also taken into account?”* [27], p. 192.

UDM have unique insights gained from their life experiences: their participation remains essential. To facilitate the participation of UDM, actors of structural compensation could use their resources to create conditions that would promote UDM participation (e.g. by providing meeting spaces, creating trust relationships). Findings showed how community-based organisations were identified as reliable partners by the UDM, but this could be the case for any organisation promoting structural compensation. As pointed by Rustage et al., several strategies can be used to overcome difficulties in partnering with UDM and facilitate collaboration, like “*outreach*,* recruitment outlets such as non-governmental organisations () and religious groups trusted by migrants*” [53], p 10. Thus, structural compensation organisations could be a facilitator to support the participation of UDM and could use their resources to take actions that would enable UDM to be more involved in various stages of an intervention.

This scoping review has some limitations. The terminology related to participatory approaches and undocumented immigration remains non-standardised [62, 63], which presents a challenge in ensuring the comprehensive inclusion of relevant literature. Consequently, additional studies employing alternative keywords could have been missed in this scoping review. The contribution of the information specialist limited this bias. We identified nine citations reporting health interventions aiming to improve healthcare access for UDM. This low number of included studies could be explained by the fact that actors of structural compensation (such as NGO) are not required to publish or have less time to devote to this task among their other missions (provide health care, advocacy, searching for funding, etc.). Additionally, this review was limited to peer-reviewed literature and did not include grey literature where more structural compensation interventions could have been found. In recent years, an increasing number of publications have arisen from NGO [64]. A systematic search of the websites of some key NGO providing health care to UDM in Europe (e.g. Doctors of the World or the Red Cross), or of the grey literature could complement this review. The limited number of included studies could also be explained by the lack of available data on UDM health, which restricts scholars’ ability to conduct further research. Furthermore, the structural compensation approach is not yet well known, and therefore not widely studied. However, as stated in Campbell et al., a scoping review is distinguished by its methodological approach, which involves the inclusion of a limited number of studies, enabling more detailed data extraction and addressing a specific focus within a broader question [65]. The findings of this scoping review were useful in identifying European countries that could be further investigated regarding participation and interventions of structural compensation for UDM. Finally, one should not underestimate the risk of a publication bias: structural compensation interventions could have a limited impact and are thus less likely to be published [66]. This study offers a substantial provision of concrete knowledge for individuals involved in the implementation of interventions aiming to improve healthcare access for UDM, including policymakers, healthcare professionals, and other relevant stakeholders. The study provides an overview of different interventions including participatory approaches that address the diverse health care needs of UDM, offering valuable insights for the design of future policies and practices. Furthermore, the review sheds light on the potential of structural compensation to address the specific challenges faced by this population. Further research is therefore required to gain a deeper understanding of the concept of structural compensation and the practices and experiences associated with it. This will enable a more appropriate response to be formulated about the use of participative approaches with UDM.

## Conclusion

Integrating participatory approaches into structural compensation interventions appears possible, even in a context of limited resources and with UDM. The integration of these approaches across all stages of an intervention may present challenges, but it should not be regarded as an absolute obstacle to their application. These insights could be crucial for those engaged in structural compensation interventions, offering guidance on the ethical challenges. Furthermore, they point to opportunities for enhancing the effectiveness of such interventions.

## Supplementary Information

Below is the link to the electronic supplementary material.

Supplementary Material 1

Supplementary Material 2

## Data Availability

No datasets were generated or analysed during the current study.
