# Supplementary material for: Structural Compensation and Participatory Approaches: A Way to Improve Access to Health Care for Undocumented Immigrants? A Scoping Review
Source: J Immigr Minor Health. 2025 Nov 18;28(4):979–89. doi: 10.1007/s10903-025-01802-x (PMC13451334; doi:10.1007/s10903-025-01802-x)
Supplement: Supplementary file 1 [file 10903_2025_1802_MOESM1_ESM.pdf]

**Supplementary Table S1** Search strategy for each selected database			
**Database**	**Search strategy**	**Date of search**	**Results**
Pubmed	*("Undocumented Immigrants"[mh] OR "undocumented migrant*"[tiab] OR "undocumented immigrant*"[tiab] OR "undocumented foreign-born"[tiab] OR "undocumented patient*"[tiab] OR "undocumented worker*"[tiab] OR "undocumented migration*"[tiab] OR "undocumented immigration*"[tiab] OR "unauthorized migrant*"[tiab] OR "unauthorized immigrant*"[tiab] OR "unauthorized foreign-born"[tiab] OR "unauthorized patient*"[tiab] OR "unauthorized worker*"[tiab] OR "unauthorized migration*"[tiab] OR "unauthorized immigration*"[tiab] OR "irregular migrant*"[tiab] OR "irregular immigrant*"[tiab] OR "irregular patient*"[tiab] OR "irregular worker*"[tiab] OR "irregular migration*"[tiab] OR "irregular immigration*"[tiab] OR "irregular labor"[tiab] OR "unregistered migrant*"[tiab] OR "unregistered patient*"[tiab] OR "unregistered worker*"[tiab] OR "illegal migrant*"[tiab] OR "illegal immigrant*"[tiab] OR "illegal worker*"[tiab] OR "illegal migration*"[tiab] OR "illegal immigration*"[tiab] OR "illegal labor"[tiab] OR "illegal labour"[tiab] OR "Undocumented Alien*"[tiab] OR "Undocumented Latino Immigrant*"[tiab] OR "Undocumented Latinx Immigrant*"[tiab] OR "Undocumented African Immigrant*"[tiab] OR "Undocumented Adult*"[tiab] OR "illegal alien*"[tiab] OR "illegal migrant worker*"[tiab] OR "undocumented migrant worker*"[tiab] OR "Undocumented Immigrant"[tiab:~2] OR "Undocumented Immigrants"[tiab:~2]) AND ("Health Services Accessibility"[mh] OR "Health Services Research"[mh] OR "Insurance Coverage"[mh] OR "Health Services Accessibility"[tiab] OR "Access to Health Services"[tiab] OR "Access to Health Care"[tiab] OR "Accessibility of Health Services"[tiab] OR "Availability of Health Services"[tiab] OR "Health Services Availability"[tiab] OR "Access to Care"[tiab] OR "Access to Cares"[tiab] OR "Program Accessibility"[tiab] OR "Access to Therapy"[tiab] OR "Access to Therapies"[tiab] OR "Access to Treatment"[tiab] OR "Access to Treatments"[tiab] OR "intervention accessibility"[tiab] OR "health access"[tiab] OR "health accessibility"[tiab] OR "healthcare access"[tiab] OR "healthcare accessibility"[tiab] OR "health care access"[tiab] OR "health care accessibility"[tiab] OR "health service access"[tiab] OR "health services access"[tiab] OR "health service access"[tiab] OR "health service accessibility"[tiab] OR "access to health care"[tiab] OR "Access to Health Service"[tiab] OR "access to healthcare"[tiab] OR "access to health"[tiab] OR "care access"[tiab] OR "care accessibility"[tiab] OR "Health Services Research"[tiab] OR "Health Care Research"[tiab] OR "Healthcare Research"[tiab] OR "Action Research"[tiab] OR "Health*	*13/06/2024*	*807*

	care coverage "[tiab] OR "Health Care Delivery"[tiab] OR "access to health insurance"[tiab] OR "Universal Health Insurance"[tiab] OR "Universal Coverage"[tiab] OR "Health insurance"[tiab] OR "Insurance Coverage"[tiab] OR "Insurance Status"[tiab] OR "accessible health care"[tiab] OR "accessible health services"[tiab] OR "accessible healthcare"[tiab] OR "health service delivery"[tiab] OR "accessing healthcare"[tiab] OR "continuum of care"[tiab] OR "health coverage"[tiab] OR "healthcare provision"[tiab] OR "health care provision"[tiab] OR "health service organization"[tiab] OR "health services organization"[tiab] OR "health service organisation"[tiab] OR "health services organisation"[tiab] OR "Program Access"[tiab] OR "Intervention Access"[tiab] OR "Therapy access"[tiab] OR "Treatment access"[tiab])		
CINAHL	(MH "Undocumented Immigrants" OR TI "undocumented migrant*" OR TI "undocumented immigrant*" OR TI "undocumented foreign-born" OR TI "undocumented patient*" OR TI "undocumented worker*" OR TI "undocumented migration*" OR TI "undocumented immigration*" OR TI "unauthorized migrant*" OR TI "unauthorized immigrant*" OR TI "unauthorized foreign-born" OR TI "unauthorized patient*" OR TI "unauthorized worker*" OR TI "unauthorized migration*" OR TI "unauthorized immigration*" OR TI "irregular migrant*" OR TI "irregular immigrant*" OR TI "irregular patient*" OR TI "irregular worker*" OR TI "irregular migration*" OR TI "irregular immigration*" OR TI "irregular labor" OR TI "unregistered migrant*" OR TI "unregistered patient*" OR TI "unregistered worker*" OR TI "illegal migrant*" OR TI "illegal immigrant*" OR TI "illegal worker*" OR TI "illegal migration*" OR TI "illegal immigration*" OR TI "illegal labor" OR TI "illegal labour" OR TI "Undocumented Alien*" OR TI "Undocumented Latino Immigrant*" OR TI "Undocumented Latinx Immigrant*" OR TI "Undocumented African Immigrant*" OR TI "Undocumented Adult*" OR TI "illegal alien*" OR TI "illegal migrant worker*" OR TI "undocumented migrant worker*" OR TI "Undocumented Immigrant" OR TI "Undocumented Immigrants" OR AB "undocumented migrant*" OR AB "undocumented immigrant*" OR AB "undocumented foreign-born" OR AB "undocumented patient*" OR AB "undocumented worker*" OR AB "undocumented migration*" OR AB "undocumented immigration*" OR AB "unauthorized migrant*" OR AB "unauthorized immigrant*" OR AB "unauthorized foreign-born" OR AB "unauthorized patient*" OR AB "unauthorized worker*" OR AB "unauthorized migration*" OR AB "unauthorized immigration*" OR AB "irregular migrant*" OR AB "irregular immigrant*" OR AB "irregular patient*" OR AB "irregular worker*" OR AB "irregular migration*" OR AB "irregular immigration*" OR AB "irregular labor" OR AB "unregistered migrant*" OR AB "unregistered patient*" OR AB "unregistered worker*" OR AB "illegal migrant*" OR AB "illegal immigrant*" OR AB "illegal worker*" OR AB "illegal migration*" OR AB "illegal immigration*" OR AB "illegal labor" OR AB "illegal labour" OR AB "Undocumented Alien*" OR AB	*13/06/2024*	*763*

	"Undocumented Latino Immigrant*" OR AB "Undocumented Latinx Immigrant*" OR AB "Undocumented African Immigrant*" OR AB "Undocumented Adult*" OR AB "illegal alien*" OR AB "illegal migrant worker*" OR AB "undocumented migrant worker*" OR AB "Undocumented Immigrant" OR AB "Undocumented Immigrants") AND (MH"Health Services Accessibility+" OR MH"Health Care Delivery+" OR MH"Health Services +" OR MH"Health Services Research+" OR MH "Insurance Coverage" OR TI "Health Services Accessibility" OR TI "Access to Health Services" OR TI "Access to Health Care" OR TI "Accessibility of Health Services" OR TI "Availability of Health Services" OR TI "Health Services Availability" OR TI "Access to Care" OR TI "Access to Cares" OR TI "Program Accessibility" OR TI "Access to Therapy" OR TI "Access to Therapies" OR TI "Access to Treatment" OR TI "Access to Treatments" OR TI "intervention accessibility" OR TI "health access" OR TI "health accessibility" OR TI "healthcare access" OR TI "healthcare accessibility" OR TI "health care access" OR TI "health care accessibility" OR TI "health service access" OR TI "health services access" OR TI "health service access" OR TI "health service accessibility" OR TI "access to health care" OR TI "Access to Health Service" OR TI "access to healthcare" OR TI "access to health" OR TI "care access" OR TI "care accessibility" OR TI "Health Services Research" OR TI "Health Care Research" OR TI "Healthcare Research" OR TI "Action Research" OR TI "Health care coverage " OR TI "Health Care Delivery" OR TI "access to health insurance" OR TI "Universal Health Insurance" OR TI "Universal Coverage" OR TI "Health insurance" OR TI "Insurance Coverage" OR TI "Insurance Status" OR TI "accessible health care" OR TI "accessible health services" OR TI "accessible healthcare" OR TI "health service delivery" OR TI "accessing healthcare" OR TI "continuum of care" OR TI "health coverage" OR TI "healthcare provision" OR TI "health care provision" OR TI "health service organization" OR TI "health services organization" OR TI "health service organisation" OR TI "health services organisation" OR TI "Program Access" OR TI "Intervention Access" OR TI "Therapy access" OR TI "Treatment access" OR AB "Health Services Accessibility" OR AB "Access to Health Services" OR AB "Access to Health Care" OR AB "Accessibility of Health Services" OR AB "Availability of Health Services" OR AB "Health Services Availability" OR AB "Access to Care" OR AB "Access to Cares" OR AB "Program Accessibility" OR AB "Access to Therapy" OR AB "Access to Therapies" OR AB "Access to Treatment" OR AB "Access to Treatments" OR AB "intervention accessibility" OR AB "health access" OR AB "health accessibility" OR AB "healthcare access" OR AB "healthcare accessibility" OR AB "health care access" OR AB "health care accessibility" OR AB "health service access" OR AB "health services access" OR AB "health service access" OR AB "health service accessibility" OR AB "access to health care" OR AB "Access to Health Service" OR AB "access to healthcare" OR AB "access to health" OR AB "care access" OR AB "care accessibility" OR AB "Health Care Research" OR AB "Healthcare Research" OR AB "Action Research" OR AB "Health care coverage " OR AB "Health Care Delivery" OR AB "access to health insurance" OR AB "Universal Health Insurance" OR AB "Universal Coverage" OR AB "Health insurance" OR AB "Insurance Coverage" OR AB "Insurance Status" OR AB "accessible health care" OR		

	AB "accessible health services" OR AB "accessible healthcare" OR AB "health service delivery" OR AB "accessing healthcare" OR AB "continuum of care" OR AB "health coverage" OR AB "healthcare provision" OR AB "health care provision" OR AB "health service organization" OR AB "health services organization" OR AB "health service organisation" OR AB "health services organisation" OR AB "Program Access" OR AB "Intervention Access" OR AB "Therapy access" OR AB "Treatment access")		
Scopus	(TITLE-ABS-KEY ({Undocumented Immigrants}) OR TITLE-ABS ("undocumented migrant*") OR TITLE-ABS ("undocumented immigrant*") OR TITLE-ABS ({undocumented foreign-born}) OR TITLE-ABS ("undocumented patient*") OR TITLE-ABS ("undocumented worker*") OR TITLE-ABS ("undocumented migration*") OR TITLE-ABS ("undocumented immigration*") OR TITLE-ABS ("unauthorized migrant*") OR TITLE-ABS ("unauthorized immigrant*") OR TITLE-ABS ({unauthorized foreign-born}) OR TITLE-ABS ("unauthorized patient*") OR TITLE-ABS ("unauthorized worker*") OR TITLE-ABS ("unauthorized migration*") OR TITLE-ABS ("unauthorized immigration*") OR TITLE-ABS ("irregular migrant*") OR TITLE-ABS ("irregular immigrant*") OR TITLE-ABS ("irregular patient*") OR TITLE-ABS ("irregular worker*") OR TITLE-ABS ("irregular migration*") OR TITLE-ABS ("irregular immigration*") OR TITLE-ABS ({irregular labor}) OR TITLE-ABS ("unregistered migrant*") OR TITLE-ABS ("unregistered patient*") OR TITLE-ABS ("unregistered worker*") OR TITLE-ABS ("illegal migrant*") OR TITLE-ABS ("illegal immigrant*") OR TITLE-ABS ("illegal worker*") OR TITLE-ABS ("illegal migration*") OR TITLE-ABS ("illegal immigration*") OR TITLE-ABS ({illegal labor}) OR TITLE-ABS ({illegal labour}) OR TITLE-ABS ("Undocumented Alien*") OR TITLE-ABS ("Undocumented Latino Immigrant*") OR TITLE-ABS ("Undocumented Latinx Immigrant*") OR TITLE-ABS ("Undocumented African Immigrant*") OR TITLE-ABS ("Undocumented Adult*") OR TITLE-ABS ("illegal alien*") OR TITLE-ABS ("illegal migrant worker*") OR TITLE-ABS ("undocumented migrant worker*") OR TITLE-ABS ({Undocumented Immigrant}) OR TITLE-ABS ({Undocumented Immigrants})) AND (TITLE-ABS-KEY ({Health Services Accessibility}) OR TITLE-ABS-KEY ({Health Services Research}) OR TITLE-ABS-KEY ({Insurance Coverage}) OR TITLE-ABS ({Health Services Accessibility}) OR TITLE-ABS ({Access to Health Services}) OR TITLE-ABS ({Access to Health Care}) OR TITLE-ABS ({Accessibility of Health Services}) OR TITLE-ABS ({Availability of Health Services}) OR TITLE-ABS ({Health Services Availability}) OR TITLE-ABS ({Access to Care}) OR TITLE-ABS ({Access to Cares}) OR TITLE-ABS ({Program Accessibility}) OR TITLE-ABS ({Access to Therapy}) OR TITLE-ABS ({Access to Therapies}) OR TITLE-ABS ({Access to Treatment}) OR TITLE-ABS ({Access to Treatments}) OR TITLE-ABS ({intervention accessibility}) OR TITLE-ABS ({health access}) OR TITLE-ABS ({health accessibility}) OR TITLE-ABS ({healthcare access}) OR TITLE-ABS ({healthcare accessibility}) OR TITLE-ABS ({health care access}) OR TITLE-ABS ({health care accessibility}) OR TITLE-ABS ({health service access}) OR TITLE-ABS ({health services access}) OR TITLE-ABS ({health service access}) OR TITLE-ABS ({health service accessibility})	*13/06/2024*	*844*

	OR TITLE-ABS ({access to health care}) OR TITLE-ABS ({Access to Health Service}) OR TITLE-ABS ({access to healthcare}) OR TITLE-ABS ({access to health}) OR TITLE-ABS ({care access}) OR TITLE-ABS ({care accessibility}) OR TITLE-ABS ({Health Services Research}) OR TITLE-ABS ({Health Care Research}) OR TITLE-ABS ({Healthcare Research}) OR TITLE-ABS ({Action Research}) OR TITLE-ABS ({Health care coverage }) OR TITLE-ABS ({Health Care Delivery}) OR TITLE-ABS ({access to health insurance}) OR TITLE-ABS ({Universal Health Insurance}) OR TITLE-ABS ({Universal Coverage}) OR TITLE-ABS ({Health insurance}) OR TITLE-ABS ({Insurance Coverage}) OR TITLE-ABS ({Insurance Status}) OR TITLE-ABS ({accessible health care}) OR TITLE-ABS ({accessible health services}) OR TITLE-ABS ({accessible healthcare}) OR TITLE-ABS ({health service delivery}) OR TITLE-ABS ({accessing healthcare}) OR TITLE-ABS ({continuum of care}) OR TITLE-ABS ({health coverage}) OR TITLE-ABS ({healthcare provision}) OR TITLE-ABS ({health care provision}) OR TITLE-ABS ({health service organization}) OR TITLE-ABS ({health services organization}) OR TITLE-ABS ({health service organisation}) OR TITLE-ABS ({health services organisation}) OR TITLE-ABS ({Program Access}) OR TITLE-ABS ({Intervention Access}) OR TITLE-ABS ({Therapy access}) OR TITLE-ABS ({Treatment access}))		
Embase	('undocumented immigrant'/exp OR 'undocumented migrant*':ti,ab OR 'undocumented immigrant*':ti,ab OR 'undocumented foreign-born':ti,ab OR 'undocumented patient*':ti,ab OR 'undocumented worker*':ti,ab OR 'undocumented migration*':ti,ab OR 'undocumented immigration*':ti,ab OR 'unauthorized migrant*':ti,ab OR 'unauthorized immigrant*':ti,ab OR 'unauthorized foreign-born':ti,ab OR 'unauthorized patient*':ti,ab OR 'unauthorized worker*':ti,ab OR 'unauthorized migration*':ti,ab OR 'unauthorized immigration*':ti,ab OR 'irregular migrant*':ti,ab OR 'irregular immigrant*':ti,ab OR 'irregular patient*':ti,ab OR 'irregular worker*':ti,ab OR 'irregular migration*':ti,ab OR 'irregular immigration*':ti,ab OR 'irregular labor':ti,ab OR 'unregistered migrant*':ti,ab OR 'unregistered patient*':ti,ab OR 'unregistered worker*':ti,ab OR 'illegal migrant*':ti,ab OR 'illegal immigrant*':ti,ab OR 'illegal worker*':ti,ab OR 'illegal migration*':ti,ab OR 'illegal immigration*':ti,ab OR 'illegal labor':ti,ab OR 'illegal labour':ti,ab OR 'undocumented alien*':ti,ab OR 'undocumented latino immigrant*':ti,ab OR 'undocumented latinx immigrant*':ti,ab OR 'undocumented african immigrant*':ti,ab OR 'undocumented adult*':ti,ab OR 'illegal alien*':ti,ab OR 'illegal migrant worker*':ti,ab OR 'undocumented migrant worker*':ti,ab OR 'undocumented immigrant':ti,ab OR 'undocumented immigrants':ti,ab) AND ('health care access'/exp OR 'insurance'/exp OR 'health services accessibility':ti,ab OR 'access to health services':ti,ab OR 'accessibility of health services':ti,ab OR 'availability of health services':ti,ab OR 'health services availability':ti,ab OR 'access to care':ti,ab OR 'access to cares':ti,ab OR 'program accessibility':ti,ab OR 'access to therapy':ti,ab OR 'access to therapies':ti,ab OR 'access to treatment':ti,ab OR 'access to treatments':ti,ab OR 'intervention accessibility':ti,ab OR 'health access':ti,ab OR 'health accessibility':ti,ab OR 'healthcare access':ti,ab OR 'healthcare accessibility':ti,ab OR 'health care	*14/06/2024*	*864*

	access':ti,ab OR 'health care accessibility':ti,ab OR 'health services access':ti,ab OR 'health service access':ti,ab OR 'health service accessibility':ti,ab OR 'access to health care':ti,ab OR 'access to health service':ti,ab OR 'access to healthcare':ti,ab OR 'access to health':ti,ab OR 'care access':ti,ab OR 'care accessibility':ti,ab OR 'health services research':ti,ab OR 'health care research':ti,ab OR 'healthcare research':ti,ab OR 'action research':ti,ab OR 'health care coverage':ti,ab OR 'health care delivery':ti,ab OR 'access to health insurance':ti,ab OR 'universal health insurance':ti,ab OR 'universal coverage':ti,ab OR 'health insurance':ti,ab OR 'insurance coverage':ti,ab OR 'insurance status':ti,ab OR 'accessible health care':ti,ab OR 'accessible health services':ti,ab OR 'accessible healthcare':ti,ab OR 'health service delivery':ti,ab OR 'accessing healthcare':ti,ab OR 'continuum of care':ti,ab OR 'health coverage':ti,ab OR 'healthcare provision':ti,ab OR 'health care provision':ti,ab OR 'health service organization':ti,ab OR 'health services organization':ti,ab OR 'health service organisation':ti,ab OR 'health services organisation':ti,ab OR 'program access':ti,ab OR 'intervention access':ti,ab OR 'therapy access':ti,ab OR 'treatment access':ti,ab)<br>('undocumented immigrant'/exp OR 'undocumented migrant*':ti,ab OR 'undocumented immigrant*':ti,ab OR 'undocumented foreign-born':ti,ab OR 'undocumented patient*':ti,ab OR 'undocumented worker*':ti,ab OR 'undocumented migration*':ti,ab OR 'undocumented immigration*':ti,ab OR 'unauthorized migrant*':ti,ab OR 'unauthorized immigrant*':ti,ab OR 'unauthorized foreign-born':ti,ab OR 'unauthorized patient*':ti,ab OR 'unauthorized worker*':ti,ab OR 'unauthorized migration*':ti,ab OR 'unauthorized immigration*':ti,ab OR 'irregular migrant*':ti,ab OR 'irregular immigrant*':ti,ab OR 'irregular patient*':ti,ab OR 'irregular worker*':ti,ab OR 'irregular migration*':ti,ab OR 'irregular immigration*':ti,ab OR 'irregular labor':ti,ab OR 'unregistered migrant*':ti,ab OR 'unregistered patient*':ti,ab OR 'unregistered worker*':ti,ab OR 'illegal migrant*':ti,ab OR 'illegal immigrant*':ti,ab OR 'illegal worker*':ti,ab OR 'illegal migration*':ti,ab OR 'illegal immigration*':ti,ab OR 'illegal labor':ti,ab OR 'illegal labour':ti,ab OR 'undocumented alien*':ti,ab OR 'undocumented latino immigrant*':ti,ab OR 'undocumented latinx immigrant*':ti,ab OR 'undocumented african immigrant*':ti,ab OR 'undocumented adult*':ti,ab OR 'illegal alien*':ti,ab OR 'illegal migrant worker*':ti,ab OR 'undocumented migrant worker*':ti,ab OR 'undocumented immigrant':ti,ab OR 'undocumented immigrants':ti,ab) AND ('health care access'/exp OR 'insurance'/exp OR 'health services accessibility':ti,ab OR 'access to health services':ti,ab OR 'accessibility of health services':ti,ab OR 'availability of health services':ti,ab OR 'health services availability':ti,ab OR 'access to care':ti,ab OR 'access to cares':ti,ab OR 'program accessibility':ti,ab OR 'access to therapy':ti,ab OR 'access to therapies':ti,ab OR 'access to treatment':ti,ab OR 'access to treatments':ti,ab OR 'intervention accessibility':ti,ab OR 'health access':ti,ab OR 'health accessibility':ti,ab OR 'healthcare access':ti,ab OR 'healthcare accessibility':ti,ab OR 'health care access':ti,ab OR 'health care accessibility':ti,ab OR 'health services access':ti,ab OR 'health service access':ti,ab OR 'health service accessibility':ti,ab OR 'access to health care':ti,ab OR 'access to health service':ti,ab OR		

	'access to healthcare':ti,ab OR 'access to health':ti,ab OR 'care access':ti,ab OR 'care accessibility':ti,ab OR 'health services research':ti,ab OR 'health care research':ti,ab OR 'healthcare research':ti,ab OR 'action research':ti,ab OR 'health care coverage':ti,ab OR 'health care delivery':ti,ab OR 'access to health insurance':ti,ab OR 'universal health insurance':ti,ab OR 'universal coverage':ti,ab OR 'health insurance':ti,ab OR 'insurance coverage':ti,ab OR 'insurance status':ti,ab OR 'accessible health care':ti,ab OR 'accessible health services':ti,ab OR 'accessible healthcare':ti,ab OR 'health service delivery':ti,ab OR 'accessing healthcare':ti,ab OR 'continuum of care':ti,ab OR 'health coverage':ti,ab OR 'healthcare provision':ti,ab OR 'health care provision':ti,ab OR 'health service organization':ti,ab OR 'health services organization':ti,ab OR 'health service organisation':ti,ab OR 'health services organisation':ti,ab OR 'program access':ti,ab OR 'intervention access':ti,ab OR 'therapy access':ti,ab OR 'treatment access':ti,ab)		
