# Supplementary material for: Structural Compensation and Participatory Approaches: A Way to Improve Access to Health Care for Undocumented Immigrants? A Scoping Review
Source: J Immigr Minor Health. 2025 Nov 18;28(4):979–89. doi: 10.1007/s10903-025-01802-x (PMC13451334; doi:10.1007/s10903-025-01802-x)
Supplement: Supplementary file 2 [file 10903_2025_1802_MOESM2_ESM.pdf]

# Supplementary file S2 The list of topics investigated for the data extraction

# General information

- Authors
- Title
- Year
- Country
- Discipline
- Journal name
- Objective of the study
- Citation
- Type of studies
- Methods of data collection

# Target group

- Only UDM (yes/no)
- Other recipients
- Specific health issue (yes/no)
- Type of health issue

### Social Health Determinants (PROGRESS +)

- Place of residence
- Ethnie
- Occupational status
- Gender
- Adults
- Children (≤18)
- Elderly people (≥65)
- Religion
- Education level
- Socioeconomic status
- Social capital

- Others Social Health Determinants

# Health access

- Issue of structural access to healthcare
- Issue of "Acceptability" (yes/no)
- Issue of " availability"(yes/no)
- Issue of "Accessibility"(yes/no)
- Issue of "Accommodation"(yes/no)
- Issue of "Affordability"(yes/no)
- Other issue of health access (yes/no)

# Intervention

**Description intervention**

- Intervention content
- Location
- Recipient of the intervention
- User of the intervention
- Fundings
- Who requests the intervention?
- Which health issue is identified?
- How the issue is defined?
- Who has defined the issue? (users, recipients, funder, requester)
- Health access dimensions targeted by the intervention (affordability, availability, accessibility, accommodation, acceptability)
- Others dimension
- Values tied to the intervention

**Implementation**

- Leadership
- Coordinator
- Execution on the ground

**Level of development**

- Micro level (patient-provider)
- Meso level (service level)

- Macro level (policies & legislations)

**Evaluation**

- Indicators used
- Results about health access
- Other results
- (Improvement of) integration of recipients into the healthcare system (yes/no)
- If improvement: how?
- Collaboration with the healthcare system (yes/no)
- If collaboration: how?

**Recommendations**

- Micro level (patient-provider)
- Meso level (service level)
- Macro level (policies & legislations)
- Comments
